# S‐Nitrosylation of Akt by organic nitrate delays revascularization and the recovery of cardiac function in mice following myocardial infarction

**DOI:** 10.1111/jcmm.15263

**Published:** 2020-10-30

**Authors:** Xiao‐Yan Li, Hong‐Ming Zhang, Gui‐Peng An, Mo‐Yan Liu, Shu‐Fang Han, Qun Jin, Ying Song, Yi‐Meng Lin, Bo Dong, Shuang‐Xi Wang, Ling‐Bo Meng

**Affiliations:** ^1^ Department of Cardiology the 960^th^Hospital of Chinese People's Liberation Army Jinan China; ^2^ Department of Cardiology Qilu Hospital of Shandong University Jinan China; ^3^ Department of Cardiology Shandong Provincial Hospital Shandong University Jinan China; ^4^ Department of Cardiology The Second Hospital affiliated to Harbin Medical University Harbin China

**Keywords:** Akt, angiogenesis, myocardial infarction, organic nitrate, S‐Nitrosylation

## Abstract

The effects of long‐term nitrate therapy are compromised due to protein S‐Nitrosylation, which is mediated by nitric oxide (NO). This study is to determine the role of Akt S‐Nitrosylation in the recovery of heart functions after ischaemia. In recombinant Akt protein and in HEK293 cells, NO donor decreased Akt activity and induced Akt S‐Nitrosylation, but was abolished if Akt protein was mutated by replacing cysteine 296/344 with alanine (Akt‐C296/344A). In endothelial cells, NO induced Akt S‐Nitrosylation, reduced Akt activity and damaged multiple cellular functions including proliferation, migration and tube formation. These alterations were ablated if cells expressed Akt‐C296/344A mutant. In *Apoe^−/−^* mice, nitroglycerine infusion increased both Akt S‐Nitrosylation and infarct size, reduced Akt activity and capillary density, and delayed the recovery of cardiac function in ischaemic hearts, compared with mice infused with vehicle. Importantly, these in vivo effects of nitroglycerine in *Apoe^−/−^* mice were remarkably prevented by adenovirus‐mediated enforced expression of Akt‐C296/344A mutant. In conclusion, long‐term usage of organic nitrate may inactivate Akt to delay ischaemia‐induced revascularization and the recovery of cardiac function through NO‐mediated S‐Nitrosylation.

## 1 INTRODUCTION

Organic nitrates are still widely used in the therapy of patients with ischaemic heart disease because they induce vasorelaxation by releasing nitric oxide (NO) to activate soluble guanylyl cyclase to increase the levels of cyclic guanosine monophosphate in vascular smooth muscle cells.^1^, ^2^ However, continuous exposure to nitrate esters can lead to nitrate tolerance. Whether long‐term nitrate therapy delays the recovery of cardiac function after acute myocardial infarction (MI) remains unknown.

In eukaryotic cells, regulation of protein properties and function by post‐translational modification is the central molecular mechanism that mediates signal transduction. S‐nitrosylation is a ubiquitous redox‐related modification of cysteine thiol by NO, which transduces NO bioactivity.^3^, ^4^ Accumulating evidence suggests that the S‐nitrosylated products play key roles in human health and diseases.^5^, ^6^ For example, nitrate therapy may S‐nitrosylate some proteins, such as soluble guanylyl cyclase, endothelial NO synthase and prostacyclin synthase, resulting in oxidative stress, endothelial dysfunction and nitrate tolerance.^7^, ^8^, ^9^, ^10^, ^11^

Akt is a serine/threonine kinase, which contains multiple cysteines, and regulates essential cellular functions including survival, proliferation, metabolism and patterned gene expression in vascular homeostasis and angiogenesis.^12^ Many angiogenic functions attributed to vascular endothelial growth factor are mediated by multiple intracellular signalling such as Akt and AMP‐activated protein kinase.^13^, ^14^We have reported that Akt inactivation is involved in the impaired ischaemia‐induced angiogenesis in diabetes by increasing intracellular pH value.^15^ Previous studies have shown that Akt can be nitrosylated resulting in decreased activity after burn injury,^16^ in insulin resistance^17^ and ischaemic brain.^18^ Whether Akt S‐nitrosylation by NO contributes to the delayed recovery of cardiac function in mice following MI is poorly understood.

Based on these observations, we hypothesized that long‐term nitrate therapy may delay the recovery of cardiac function after MI through Akt S‐nitrosylation. Our results revealed that Akt was inactivated through NO‐mediated S‐nitrosylation in endothelial cells, and inhibition of Akt S‐nitrosylation promotes angiogenesis and improves the recovery of heart functions in mice treated with nitroglycerin (NTG) following MI. Clinically, inhibition of Akt S‐nitrosylation is a potential effective approach to promote revascularization in patients with ischaemic heart diseases.

## 2 MATERIALS AND METHODS

An expanded section of Materials and Methods is available in the Online Data S1.

### 2.1 Reagents

Polyclonal or monoclonal antibodies against Akt, pGSK and CD31 were obtained from Cell Signaling Company. Lipofectamine^TM^ Max was from Invitrogen. The kit of Akt activity assay was from Cell Signaling Company. All drug concentrations were expressed as the final molar concentration in the buffer.

### 2.2 Animals and NTG infusion

*Apoe^−/−^* mice (8‐12 weeks old, 25 ± 5 g) were purchased from Hua‐Fu‐Kang Animal Company. All animals were housed in temperature‐controlled cages with a 12‐hour light‐dark cycle. Mice were continuously infused with NTG (50 mg/kg/day, 14 days) by planting Alzet osmotic pumps as described previously.^19^, ^20^ This study was carried out in strict accordance with the recommendations in the Guide for the Care and Use of Laboratory Animals of the National Institutes of Health. The animal protocols were reviewed and approved by the Animal Care and Use Committees of General Hospital of Jinan Military District.

### 2.3 Induction of MI

This minimally invasive method including identification of left anterior descending coronary artery (LADCA), the transthoracic puncture and ligation was performed with the assistant of ultrasound as described previously.^21^

### 2.4 Capillary density

As described previously,^22^ immunohistochemistry (IHC) was performed to assess capillary density in ischaemic heart on the 14th post‐operative day. Briefly, formalin‐fixed heart tissue was stained with antibodies against CD31. The numbers of CD31^+^ per scope were counted to represent capillary densities.

### 2.5 Echocardiography

Echocardiography with standard parasternal and apical views was conducted in the left lateral recumbent position.^23^ Systolic or diastolic left ventricular internal diameter (sLVID or dLVID), ejection fraction (EF) and fractional shortening (FS) were calculated.

### 2.6 Determinations of Akt S‐nitrosylation and activity

Proteins were extracted according to the manufacturer's specification S‐Nitrosylated Protein Detection Assay Kit (Cayman, USA) which is based on the ‘Biotin‐switch’ method as described previously.^11^ Akt activity was determined in a kinase reaction using recombinant GSK‐3α as substrate.^24^ Two µl GSK‐3α protein/ATP mixture was added into 50 µl kinase buffer and incubated at 30°C for 14 hours. Phosphorylation of the GSK‐3α can be analysed by Western blot analysis using the phospho‐GSK‐3α‐specific antibody. The level of phospho‐GSK‐3α was calculated to represent Akt activity.

### 2.7 Cell cultures

Human umbilical vein endothelial cells (HUVECs) were purchased from Cascade Biologics (Portland, OR) and grown in endothelial basal medium (Clonetics Inc Walkersville, MD) supplemented with 2% foetal calf serum (FCS) and growth factors, penicillin (100 u/mL), and streptomycin (100 µg/mL). All cells were incubated at 37°C in a humidified atmosphere of 5% CO_2_ and 95% air. Cells were grown to 70%‐80% confluent with starvation before being treated with different agents.

### 2.8 Generation of DNA construct and adenovirus infection to cells or mice

WT‐Akt cDNA was purchased from OriGene Company. All cysteine residues were replaced with alanine by using the QuikChange kit (Stratagene), according to the manufacturer's instructions as we described previously.^20^

### 2.9 Western blot

Cells or tissues were homogenized on ice in cell‐lysis buffer containing 20 mM Tris‐HCl (pH 7.5), 150 mM NaCl, 1 mM Na_2_EDTA, 1 mM EGTA, 1% Triton, 2.5 mM sodium pyrophosphate, 1 mM beta‐glycerophosphate, 1 mM Na_3_VO_4_, 1 µg/mL leupeptin and 1 mM PMSF. Protein samples were solubilized in SDS sample buffer, and 20 µg of protein was separated by SDS‐PAGE using 8%‐10% polyacrylamide gels, transferred to nitrocellulose membranes followed by incubating membrane with primary antibody and secondary antibody. Bound antibodies were detected with ECL‐enhanced chemiluminescence (Amersham Biosciences) according to the manufacturer's protocols as described previously.^25^

### 2.10 In vitro tube formation assay

The tube formation was performed as the method described previously.^26^ Cultured HUVECs were seeded on cell culture dishes coated with growth factor reduced Matrigel (BD Biosciences) and cultured in MCDB 133 medium containing 0.5% FCS with or without HG. After 24 hours, the medium was removed and the cells were fixed with 4% paraformaldehyde. Photographs were taken through a microscope (Olympus). The capillary tube area was quantified per square micrometer using image analysis software (ImageJ Corporation).

### 2.11 Cell migration

Scratch test was applied to detect the migration of cells.^27^, ^28^ When the cell growth reached 80% fusion, cell digestion was inoculated into 24‐well plates with six duplicated wells for each group. A scratch was made in the well bottom by using a sterile 10‐mL spear in cultured cells. The picture of cell migration was taken at day 0 and day 4 after scratch. The migration rate was calculated by counting the distance of cell migrations.

### 2.12 Evaluation of cell proliferation

Cell proliferation was assayed by using 3‐(4,5‐dimethyl‐2‐thiazolyl)‐2,5‐diphenyl‐2‐H‐tetrazolium bromide (MTT) as described previously.^29^ Cells were grown in a clear plate according to the desired protocol. 50 µL of serum‐free media and 50 µL of MTT reagent (5 mg/mL) were added into each well. The plate was incubated at 37°C for 3 hours. Then, dimethyl sulphoxide was added to each well and the cells were left at room temperature in the dark for 2 hours. The absorbance was read at OD = 590 nm. By averaging the duplicate reading for each sample and subtracting the culture medium background from the assay reading, the amount of absorbance is proportional to cell number.

### 2.13 Statistical analysis

All quantitative results are expressed as mean ± SEM. The normal distribution of data was tested by the Kolmogorov‐Smirnov test before statistical comparisons, and the normality/equal variance was tested to determine whether ANOVA was appropriate. Multiple comparisons were analysed with a one‐way ANOVA followed by Tukey *post hoc* tests or Bonferroni *post hoc* analyses. Comparisons between two groups were analysed by unpaired Student's *t* test between two groups. Statistical analyses were conducted using GraphPad Prism 6.0 or IBM SPSS statistics 20.0. A two‐sided *P*‐value < .05 was considered significant.

## 3 RESULTS

### 3.1 NO induces Akt protein S‐nitrosylation and decreases Akt activity in vitro

**Figure 1:**
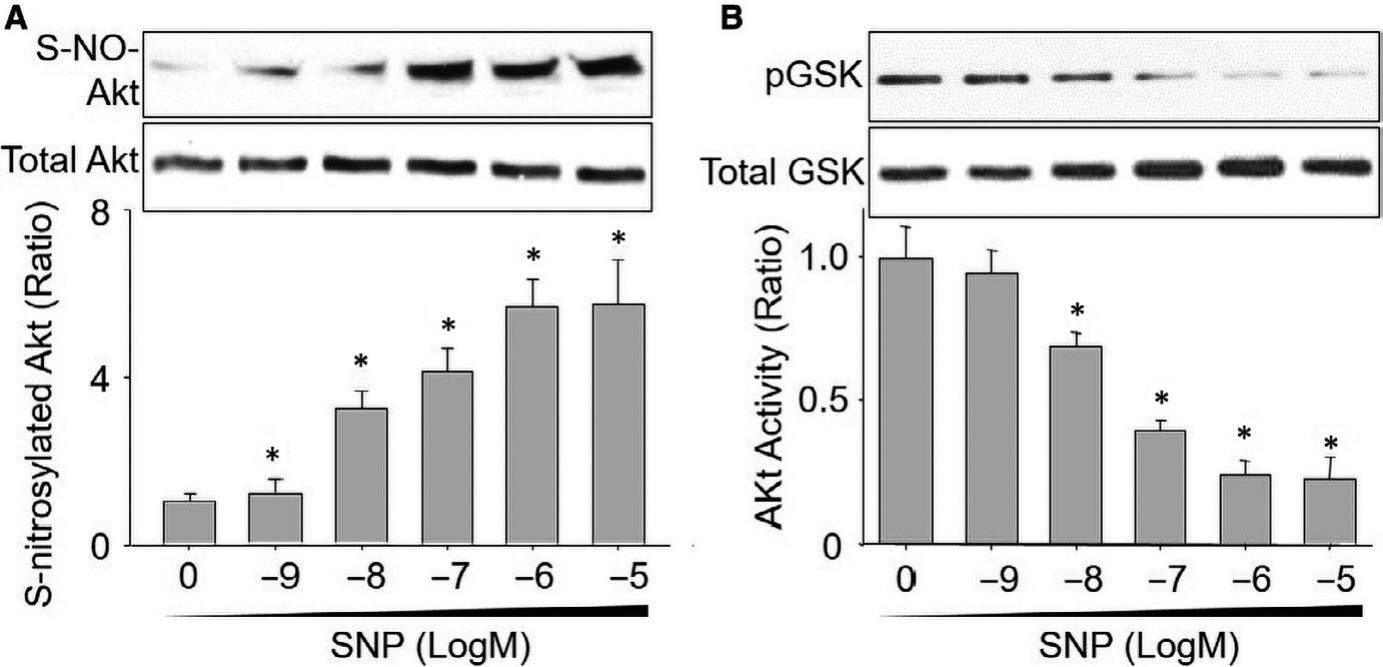
NO induces Akt protein S‐nitrosylation and decreases Akt activity in vitro. Recombinant human Akt proteins were incubated with SNP (1 × 10^−9^‐1 × 10^−5^ M) for 2 h in reaction buffer. A, S‐nitrosylated Akt (S‐NO‐Akt) was assayed by using biotin‐switch method. B, Akt activity was determined by measuring GSK3 phosphorylation. N = 3 per group. ^*^ *P* < .05 vs. vehicle (0)

NO released from organic nitrate is a highly reactive gas molecule that may cause functional dysregulations of target proteins through post‐translational modifications.^30^ We examined whether NO induces Akt S‐nitrosylation in vitro by incubating recombinant Akt protein with varying concentrations (1 × 10^−9^ to 1 × 10^−5^ M) of sodium nitroprusside (SNP), which functions as an NO donor by releasing NO directly.^31^ Akt S‐nitrosylation was assessed by using biotin‐switch method. As shown in Figure 1A, the S‐nitrosylated levels of recombinant Akt protein gradually increased at the beginning concentration of SNP at 1 × 10^−8^ M after 2‐hour incubation and reached the peak levels at 1 × 10^−6^ M. In addition, increased Akt S‐nitrosylation was associated with reduced Akt activity (Figure 1B), as determined by measuring Akt substrate GSK3 phosphorylation.^32^ Further, increasing concentrations of SNP further decreased Akt activity. SNP treatment did not alter the total levels of Akt, suggesting that SNP‐induced Akt S‐nitrosylation was not associated with the stability of Akt protein. All these data support the notion that SNP‐induced Akt S‐nitrosylation is due to a direct modification of Akt protein by NO.

### 3.2 NO S‐nitrosylates Akt protein at cysteine 296 and cysteine 344 to reduce Akt activity in HEK293 cells

**Figure 2:**
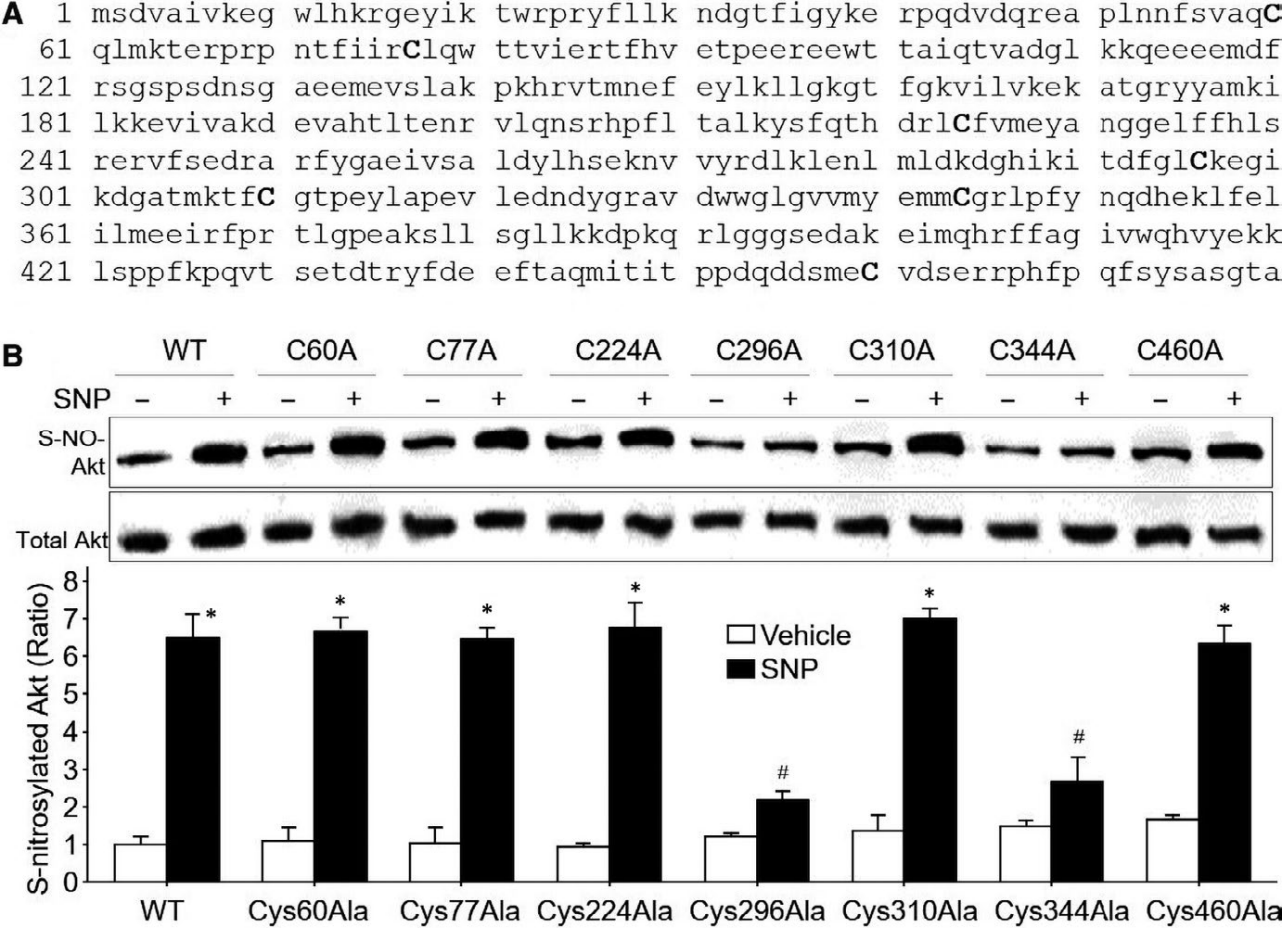
NO S‐nitrosylates Akt protein at Cys296 and Cys344 to reduce Akt activity in cells. A, Human Akt protein contains seven cysteine residues, which locate in the 60th, 77th, 224th, 296th, 310th, 344th and 460th of amino acids. B, HEK293 cells were transfected with plasmids expressing HA‐tagged site‐mutated Akt for 48 h and then treated with SNP (1 μM) for 2 h. Akt proteins in total cell lysates were purified by using anti‐HA antibody and subjected to measure Akt S‐nitrosylation by using biotin‐switch method. N = 3 per group. ^*^ *P* < .05 vs. WT plus vehicle. ^#^ *P* < .05 vs. WT plus SNP

Since S‐nitrosylation is a modification of cysteine thiol by NO,^33^ we proposed a mechanism by which NO might directly modify Akt protein at cysteine residue. To test this idea, we performed analyses of amino acid sequence in Akt protein. As shown in Figure 2A, Akt proteins contain seven cysteine residues, which locate in the 60th, 77th, 224th, 296th, 310th, 344th and 460th within human Akt protein, indicating Akt protein is able to be S‐nitrosylated by NO.

To determine which cysteine residue is the potential site of S‐nitrosylation, we generated the site‐directed mutagenesis plasmids by replacing each cysteine with alanine and transfected these plasmids into HEK293 cells following by exposure of SNP. As depicted in Figure 2B, SNP dramatically induced Akt protein S‐nitrosylation in cells transfected with plasmid expressing full‐length wild‐type Akt (WT‐Akt), compared to vehicle‐treated cells. Similarly, SNP induced Akt S‐nitrosylation in cells expressing mutated Akt if the 60th, 77th, 224th, 310th and 460th cysteine were replaced by alanine, while SNP failed to induce Akt S‐nitrosylation if cells expressed mutated Akt with replacements of the 296th and 310th cysteine by alanine. Collectively, these data demonstrate that NO induces S‐nitrosylation of Akt protein at Cys296 and Cys344 residues, partially consistent with other reports.^16^

### 3.3 SNP decreases Akt activity through NO‐mediated S‐nitrosylation in cultured human umbilicus vessel endothelial cells (HUVECs)

**Figure 3:**
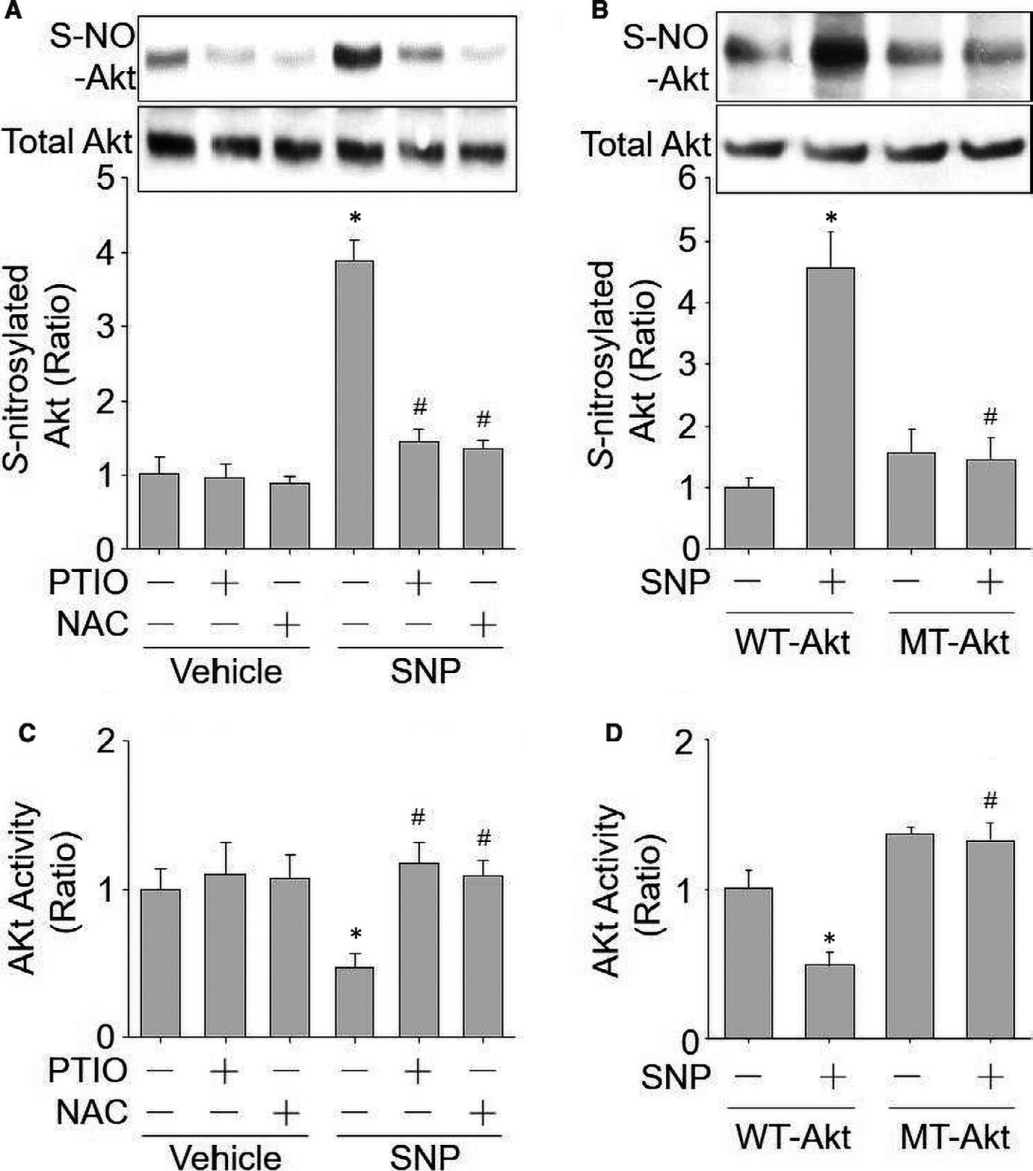
SNP decreases Akt activity through NO‐mediated S‐nitrosylation in HUVECs. (A and B) Primary HUVECs were treated with PTIO (0.3 mM) and NAC (2.0 mM) for 30 min followed by exposure of SNP (1 μM, 2 h). Total cell lysates were subjected to determine Akt S‐nitrosylation in A and Akt activity in B. N = 3 per group. ^*^ *P* < .05 vs. vehicle alone. ^#^ *P* < .05 vs. SNP alone. (C and D) HUVECs were infected with adenovirus expressing HA‐Akt‐WT or HA‐Akt‐MT (Cys296/344Ala) for 48 h followed by incubation with SNP (1 μM, 2 h). HA‐tagged Akt protein in total cell lysates was purified by using anti‐HA antibody and subjected to determine Akt S‐nitrosylation in C and Akt activity in D. N = 3 per group. ^*^ *P* < .05 vs. Akt‐WT alone. ^#^ *P* < .05 vs. Akt‐WT plus SNP

Endothelial dysfunction is an early marker for multiple cardiovascular diseases such as ischaemia‐induced angiogenesis, regulations of systemic blood pressure and vascular stiffness.^25^, ^34^, ^35^ Thus, we determined whether NO‐mediated S‐nitrosylation contributes to SNP‐induced reduction of Akt activity in endothelial cells by using either NO scavenger carboxy‐PTIO or S‐nitrosylation inhibitor N‐acetyl‐cysteine (NAC) to block NO/S‐nitrosylation signalling. In Figure 3A,B, though SNP induced Akt S‐nitrosylation and reduced Akt activity in HUVECs, both PTIO and NAC abolished Akt S‐nitrosylation and reversed Akt activity in SNP‐treated cells. These data demonstrate that SNP inhibits Akt activity through NO/S‐nitrosylation signalling.

### 3.4 SNP inhibits Akt activity in HUVECs, which depends on Akt S‐nitrosylation at Cys296 and Cys344

We next determined whether SNP decreases Akt activity through Akt S‐nitrosylation at Cys296 and Cys344. To this point, we generated adenovirus harbouring double mutants of Akt by replacing Cys296/344 to alanine (MT‐Akt). In HUVECs infected with adenovirus expressing MT‐Akt or WT‐Akt, approximate 90 per cent of Akt was exogenous Akt (GFP‐Akt fusion protein). Both WT‐Akt and endogenous Akt were able to be S‐nitrosylated, while MT‐Akt was unable to be S‐nitrosylated in endothelial cells. Therefore, MT‐Akt was called S‐nitrosylation‐resistant Akt in this study.

As depicted in Figure 3C, SNP incubation induced Akt S‐nitrosylation in HUVECs infected with adenovirus expressing WT‐Akt but not MT‐Akt. Conversely, the activity of Akt was lost more than 50% after SNP incubation in cells expressing WT‐Akt, while Akt activity was not inhibited by SNP if cells expressed S‐nitrosylation‐resistant Akt (Figure 3D). These results further support this concept that SNP‐reduced Akt activity is through Akt S‐nitrosylation at Cys296 and Cys344 in endothelial cells.

### 3.5 SNP inhibits cell proliferation through Akt S‐nitrosylation at Cys296 and Cys344 in HUVECs

**Figure 4:**
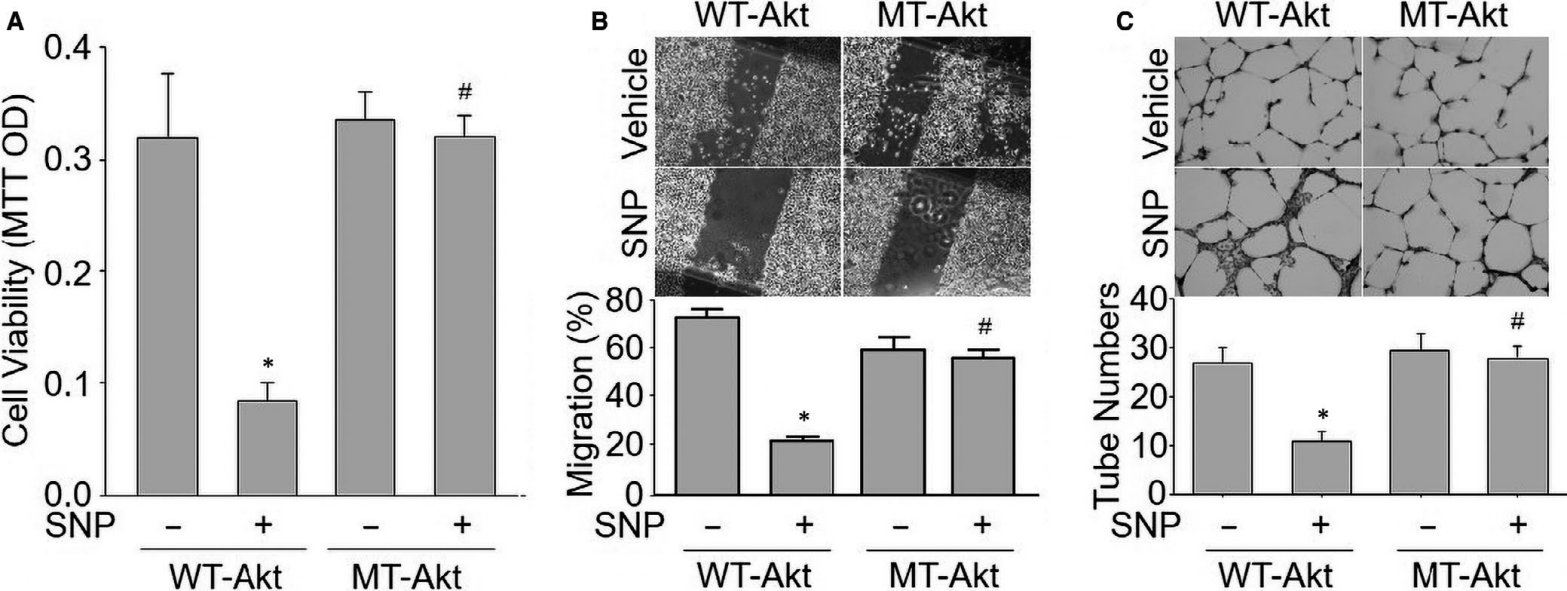
NO‐mediated Akt S‐nitrosylation inhibits cell proliferations, migrations and tube formations in HUVECs. HUVECs were infected with adenovirus expressing WT‐Akt or MT‐Akt (Cys296/344Ala) for 48 h followed by incubation with SNP (1 μM) for 2 h. A, Cell proliferation was assayed by MTT. B, Cell migration was determined by scratch test, and representative pictures were shown. Migration rate was calculated in the 3rd day after scratch. C, The representative pictures of tube formations in HUVECs were presented, and quantitative analysis was performed by calculating tube numbers per scope. N = 3 per group. ^*^ *P* < .05 vs. Akt‐WT alone. ^#^ *P* < .05 vs. Akt‐WT plus SNP

We have reported previously that Akt is a key factor to regulate cell proliferations of endothelial cells.^15^ Thus, we investigated whether SNP affected cell proliferation in cultured HUVECs through Akt S‐nitrosylation. As shown in Figure 4A, SNP dramatically inhibited cell proliferation, as detected by MTT in HUVECs expressing WT‐Akt. As hypothesized, the mutant of Akt at Cys296/344Ala abolished the SNP‐decreased cell proliferation. These data suggest that Akt S‐nitrosylation is essential for SNP‐inhibited cell proliferation in endothelial cells.

### 3.6 Mutant of Akt at Cys296 plus Cys344 to alanine abolishes SNP‐delayed cell migrations in HUVECs

Endothelial cell migration is critical to the post‐ischaemic angiogenesis.^36^ We next examined whether Akt S‐nitrosylation contributes to SNP‐inhibited cell migrations in HUVECs. As shown in Figure 4B, SNP inhibited the migration rates in HUVECs infected with adenovirus expressing WT‐Akt, but not in cells infected with adenovirus expressing mutated Akt at Cys296/344Ala (MT‐Akt). These findings, hence, in combination prove that Akt S‐nitrosylation is crucial to the cell migrations impaired by SNP.

### 3.7 SNP impairs tubulogenesis in endothelial cells via Akt S‐nitrosylation

Tube formation is a vital step in endothelial cell‐mediated angiogenesis.^37^ Therefore, we examined whether S‐nitrosylation‐resistant Akt reversed SNP‐impaired tube formation in HUVECs. As shown in Figure 4C, SNP inhibited the tube formation of HUVECs expressing WT‐Akt, while the effects of SNP on tubulogenesis were ablated by infecting cells with adenovirus expressing mutated Akt at Cys296/344Ala (MT‐Akt). In sum, these data suggest that SNP via Akt S‐nitrosylation inhibits cell proliferations, migrations and tubulogenesis in endothelial cells.

### 3.8 Long‐term NTG exposure delays the recovery of cardiac function and angiogenesis in *Apoe^−/−^* mice following MI

**Figure 5:**
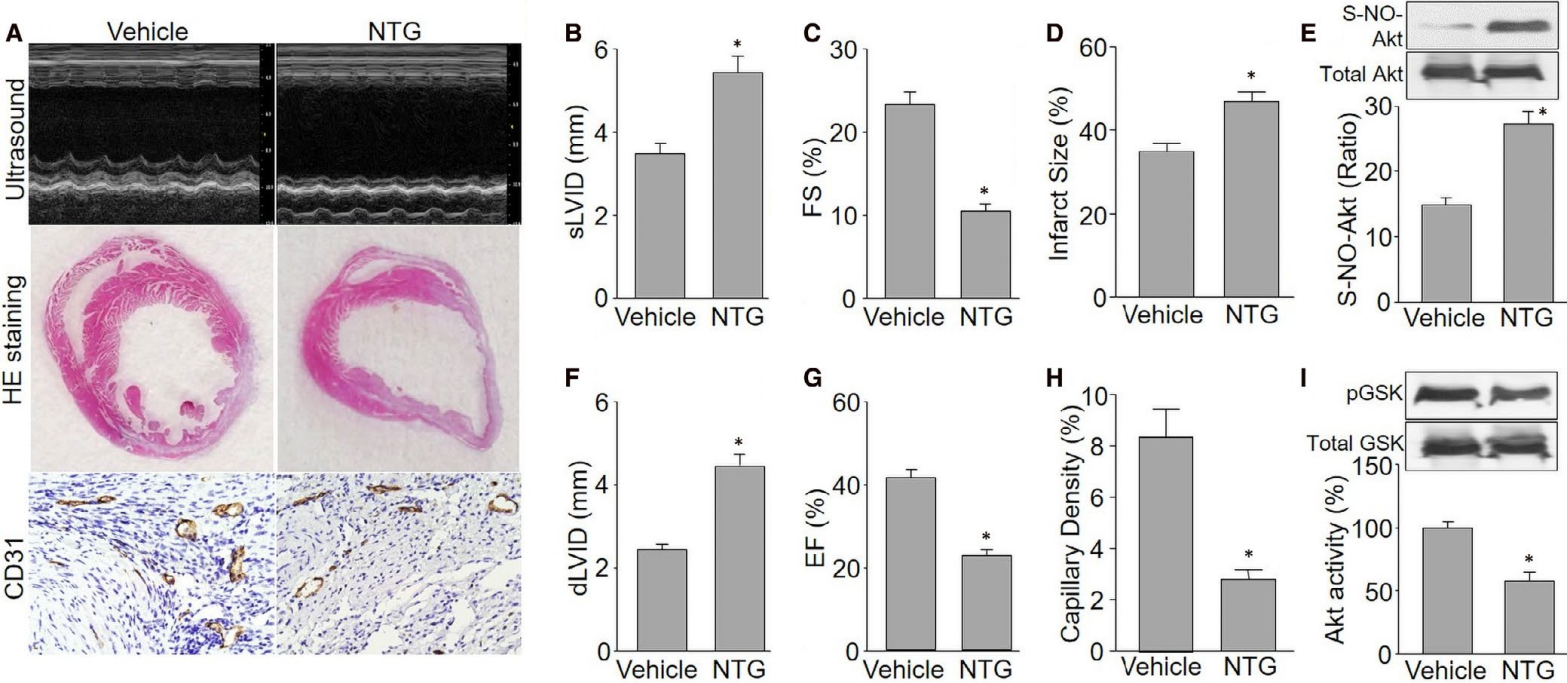
Continuous nitroglycerin (NTG) infusion induces Akt S‐nitrosylation and delays the recovery of cardiac function in *Apoe^−/−^* mice following myocardial infraction (MI). *Apoe^−/−^* mice were infused with NTG (50 mg/kg/day) for 14 d followed by MI surgery. A, Representative images showing cardiac functions by echocardiography, infarct sizes by HE staining and capillary densities by IHC analysis of CD31 in mouse hearts. B‐E, Quantitative analyses of cardiac functions were performed by calculating sLVID in B, dLVID in C, FS in D and EF in E. F, G, Quantitative analyses of infarct sizes in F and capillary densities in G were performed. H, I, Akt S‐nitrosylation in H and Akt activity in I were assayed in post‐ischaemic hearts. N is 10‐15 in each group. ^*^ *P* < .05 vs. vehicle

Next, we examined whether long‐term nitrate therapy impaired heart functions and angiogenesis in ischaemic heart. We generated in vivo model of long‐term nitrate therapy in *Apoe^−/−^* mice infused with NTG for 14 consecutive days as described previously.^2^, ^11^ MI model was established by using a minimally invasive approach in mice without thoracotomy.^21^ Heart function was examined on the 14th post‐operative day by echocardiography (Figure 5A). NTG infusion dramatically increased sLVID and dLVID, and decreased FS and EF in mice, compared with vehicle‐infused mice (Figure 5B‐E). The impaired heart functions in NTG‐infused mice were accompanied with increased infraction sizes in ischaemic hearts, as determined by HE staining (Figure 5A,F). These data demonstrate that long‐term nitrate therapy is able to delay the recovery of cardiac functions in ischaemic heart diseases.

Angiogenesis is a key regenerative event to re‐establish blood supply and repair infarcted area after MI in heart.^38^ Thus, we determined capillary densities in ischaemic hearts on the 14th post‐operative day by staining with antibody against CD31. As indicated in Figure 5A,G, NTG infusion markedly reduced the vessel density in ischaemic hearts obtained from *Apoe^−/−^* mice following MI when compared with vehicle‐treated mice.

### 3.9 NTG infusion increases Akt S‐nitrosylation and reduces Akt activity in vivo

To identify the role of Akt S‐nitrosylation in the delayed angiogenesis induced by long‐term nitrate therapy, we determined the levels of both Akt S‐nitrosylation and Akt activity in ischaemic hearts on the 14th post‐operative day. As indicated in Figure 5H, the levels of Akt S‐nitrosylation were remarkably increased in ischaemic hearts in *Apoe^−/−^* mice infused with NTG, compared to vehicle‐treated mice. Conversely, NTG reduced Akt activity in ischaemia hearts (Figure 5I). These data demonstrate that Akt protein is able to be S‐nitrosylated by long‐term nitrate therapy in vivo.

### 3.10 Long‐term nitrate therapy via Akt S‐nitrosylation inhibits post‐ischaemic angiogenesis in hearts in vivo

**Figure 6:**
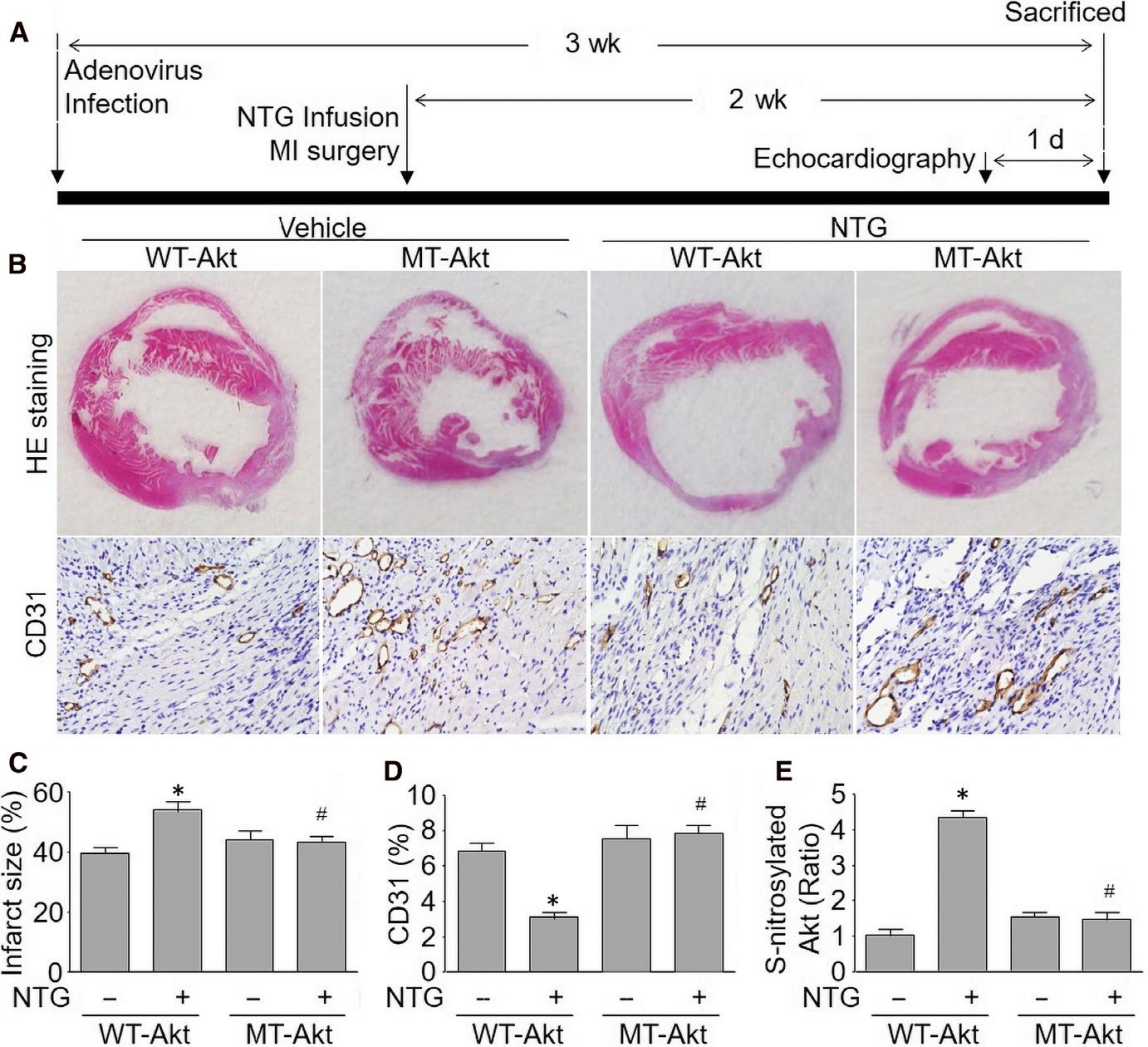
Adenovirus‐mediated exogenous expression of mutated Akt by replacing Cys296/344 with alanine promotes ischaemia‐induced angiogenesis in NTG‐infused mice. A, *Apoe^−/−^* mice were infected with adenovirus expressing WT‐Akt or MT‐Akt (Cys296/344Ala) via tail vein injection followed by 14‐day NTG infusion (50 mg/kg/day) prior to MI surgery. The graphic protocol was illustrated. B, Representative images showing HE staining and IHC analysis of CD31 in ischaemic hearts. C, D, Quantitative analyses of infarct size in C and capillary density in D were performed. E, Akt S‐nitrosylation in ischaemic heart was assayed by biotin‐switch method. N is 10‐15 in each group. ^*^ *P* < .05 vs. WT‐Akt alone. ^#^ *P* < .05 vs. WT‐Akt plus NTG

The role of Akt S‐nitrosylation in the ischaemia‐induced angiogenesis was also examined in vivo. To this end, adenovirus containing cDNA of WT‐Akt or MT‐Akt was introduced into *Apoe^−/−^* mice via tail vein injection for 1 week prior to 14‐day NTG infusion and MI surgery by LADCA ligation (Figure 6A). Continuous NTG infusion remarkably increased infraction sizes and reduced the vessel densities in ischaemic hearts obtained from NTG‐infused mice infected with adenovirus expressing cDNA of WT‐Akt (Figure 6B‐D). Vastly, capillary density exhibited a robust increase in ischaemic hearts from NTG‐infused mice expressing S‐nitrosylation‐resistant Akt (MT‐Akt). As expected, NTG failed to S‐nitrosylate Akt in mice expressing cDNA of MT‐Akt (Figure 6E). Collectively, these results reveal that Akt S‐nitrosylation is required for the angiogenic response impaired by nitrate therapy in vivo.

### 3.11 Enforced expression of S‐nitrosylation‐resistant Akt accelerates the recovery of heart function in NTG‐infused mice following MI

**Figure 7:**
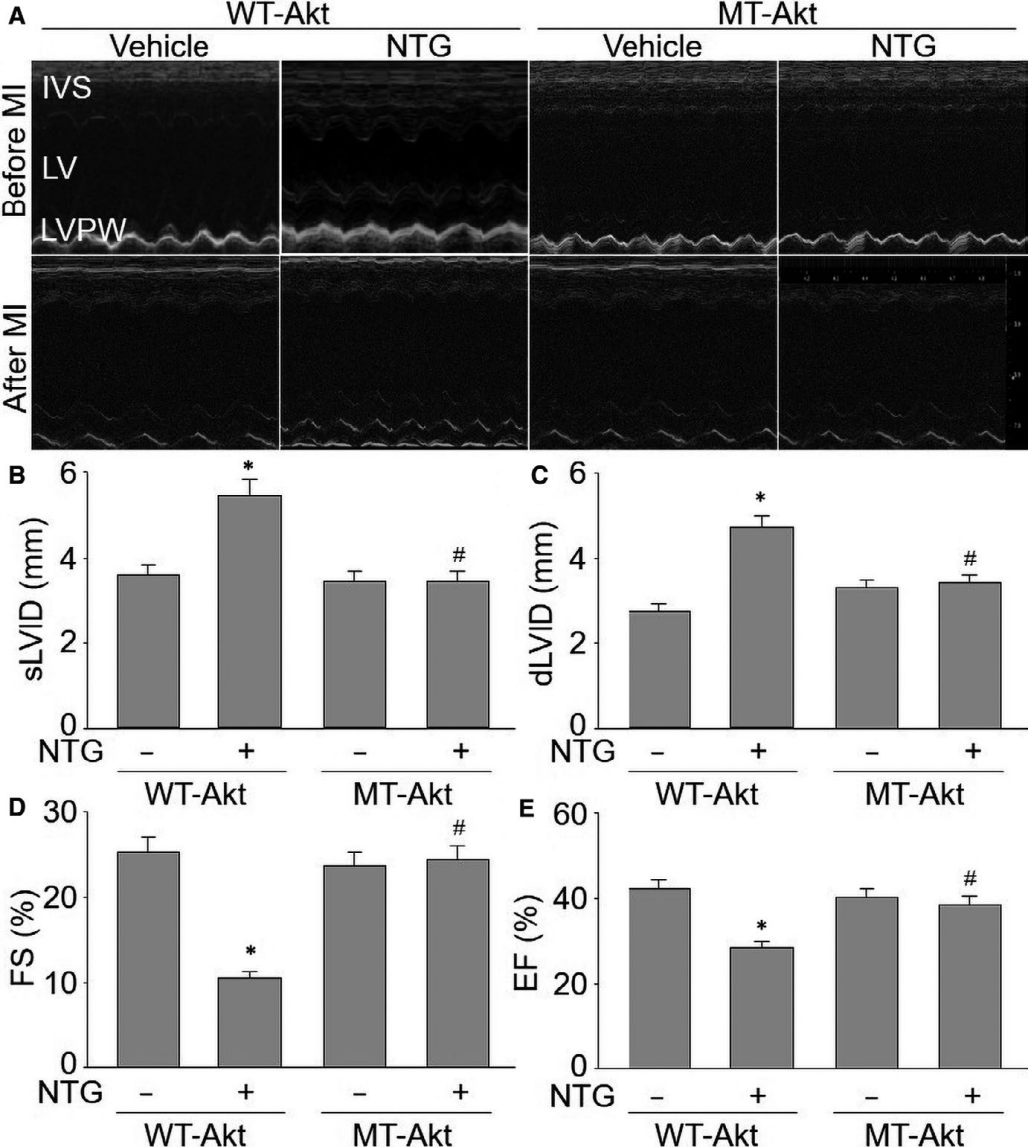
Enforced expression of S‐nitrosylation‐resistant Akt improves cardiac functions in NTG‐infused mice after heart ischaemia. The protocol of animal experiment is shown in Figure 6A. A, Representative images showing heart functions measured by echocardiography. B‐E, Quantitative analyses of cardiac functions were performed by calculating sLVID in B, dLVID in C, FS in D and EF in E. N is 10‐15 in each group. ^*^ *P* < .05 vs. WT‐Akt alone. ^#^ *P* < .05 vs. WT‐Akt plus NTG

Finally, we examined heart functions by echocardiography in *Apoe^−/−^* mice before MI surgery and two weeks after MI surgery (Figure 7A). As shown in Figure 7B‐E, the cardiac functions were comparable in four groups before MI, allowing the heart function to be evaluated after MI. However, two weeks post‐LADCA ligation, in comparison with vehicle‐treated mice, NTG increased sLVID and dLVID, and decreased FS and EF in mice expressing WT‐Akt. By contrast, NTG did not deteriorate cardiac function in mice if they positively expressed S‐nitrosylation‐resistant Akt (MT‐Akt), suggesting that Akt S‐nitrosylation contributes to the deleterious effect of nitrate therapy in global cardiac function.

## 4 DISCUSSION

In this study, we identified a novel modification of Akt protein under long‐term nitrate therapy, and this kind of post‐translational modification such as Akt S‐nitrosylation contributed to the delayed recovery of heart function after MI. Mechanically, NO derived from nitrates S‐nitrosylates Akt protein at cysteine residues and reduces Akt activity, resulting in dysfunction of endothelial cells. In this way, organic nitrates induce the side effects besides nitrate tolerance. Therefore, the current study will open new avenue to investigate the role of S‐nitrosylation in Akt functional regulation and also provide some insights to drug design for improving cardiac dysfunction in that targeting Akt S‐nitrosylation to improve the outcome of patients with MI.

The major finding of the present study is that long‐term nitrate therapy may delay the recovery of heart function after MI. Nitrate therapy has been an effective treatment for ischaemic heart disease by releasing the vasoactive principle NO,^39^ while the effects are short‐lived. In an effort to increase the duration of beneficial effects, long‐acting orally administrations of nitrates have been developed; however, patients soon develop tolerance. In such condition, patients begin losing the protective effects of nitrate therapy, which might affect the prognosis.^40^, ^41^, ^42^ In this study, we observed the new side effects of long‐term nitrate therapy as a critical factor contributing to the delayed recovery of heart function. Further, impaired angiogenesis is crucial for the delayed recovery of cardiac function because multiple functions including cell proliferation, migration and tube formation are damaged in endothelial cells when exposed to organ nitrates.

An important discovery is that Akt protein is post‐translationally modified by NO via S‐nitrosylation in endothelial cells. Previous studies have shown that Akt can be nitrosylated resulting in decreased activity in insulin resistance and ischaemic brain.^17^, ^18^ Evenly, studies have also shown that Cys296 and Cys310 are S‐nitrosylated by NO after burn injury.^16^ In this study, we further provide evidence to support the proposal that Akt is S‐nitrosylated at Cys296 and Cys344 simultaneously in endothelial cells. This evidence can be summarized as follows. First, Akt S‐nitrosylation was detectable in recombinant proteins, cells and *Apoe^−/−^* mice when treated with nitrates. Second, mutations of S‐nitrosylation sites at Cys296 and Cys344, in which NO can act by directly modifying cysteine residues on target protein, abolished Akt S‐nitrosylation both in vitro and in vivo. Till now, post‐translational modification of Akt protein has been reported to be ubiquitinated in tumorigenesis^43^ and phosphorylated by upstream kinase in insulin resistance.

Further, we linked Akt S‐nitrosylation with the impaired angiogenesis and the recovery of heart function in patient with nitrate therapy. We have previously reported that down‐regulation of Akt by intracellular pH value was involved in the delayed angiogenesis and improvement of cardiac dysfunction in diabetic heart.^15^ Angiogenesis requires angiogenic factors, such as VEGF and IGF, to stimulate vessel sprouting and remodelling of the primitive vascular network, which in turn establishes stable and functional blood vessel networks.^44^, ^45^ Akt activation severs as a common molecular mechanism by how angiogenic factors produce their effects on vascular regeneration.^46^ In this study, we identified not only a stable microenvironment but NO‐mediated signalling is essential for the neovascularization in endothelial cells. Our observations indicate that protein S‐nitrosylation is increased by nitrate therapy to cause Akt inactivation, leading to malfunctions of angiogenic factors. Though Akt signalling has been demonstrated to be important in angiogenesis,^47^, ^48^ this present study further clarifies the key role of Akt post‐translational modification such as S‐nitrosylation in angiogenesis.

## CONFLICT OF INTEREST

None.

## AUTHOR CONTRIBUTIONS

XYL, HMZ and GPA performed all experiments and drafted the manuscript. MYL, FSH, QJ, YS and YML partially performed some experiments. BD, SXW and LBM conceived the idea, designed all experiments, convinced the whole project and revised the manuscript.

## Supporting information

Supplementary Material

## Data Availability

The data sets used and analysed during the current study are available from the corresponding author on reasonable request.
