# Supplementary material for: S‐Nitrosylation of Akt by organic nitrate delays revascularization and the recovery of cardiac function in mice following myocardial infarction
Source: J Cell Mol Med. 2020 Oct 30;25(1):27–36. doi: 10.1111/jcmm.15263 (PMC7810919; doi:10.1111/jcmm.15263)
Supplement: Supplementary file 1 — Supplementary Material [file JCMM-25-27-s001.docx]

**Online Supplements**

**S-Nitrosylation of Akt by organic nitrate delays revascularization and the recovery of cardiac function in mice following myocardial infarction**

Xiao-Yan Li^1,a^, Hong-Ming Zhang^1,a^, Gui-Peng An^2,a^, Mo-Yan Liu^1^, Shu-Fang Han^1^, Qun Jin^1^, Ying Song^1^, Yi-Meng Lin^1^, Bo Dong^2,3,b^, Shuang-Xi Wang^2,b^, Ling-Bo Meng^4,b^

^1^Department of Cardiology, the 960^th^ Hospital of People's Liberation Army, Jinan, China; ^2^Department of Cardiology, Qilu Hospital of Shandong University, Jinan, China; ^3^Department of Cardiology, Shandong Provincial Hospital, Shandong University, Jinan, China; ^4^Department of Cardiology, The Second Hospital affiliated to Harbin Medical University, Harbin, China

^a^ These authors contributed equally to this work.

^b^ Correspondence to Ling-Bo Meng, Shuang-Xi Wang, and Bo Dong, Email menglingbo50@163.com

**Supplementary Methods and Materials**

**Reagents**

Polyclonal or monoclonal antibodies against Akt, pGSK, and CD31 were obtained from Cell Signaling Company. Lipofectamine^TM^ Max was from Invitrogen. The kit of Akt activity assay was from Cell Signaling Company. All drug concentrations were expressed as the final molar concentration in the buffer.

**Animals and experimental protocols**

Male *Apoe^-/-^* mice (8-12 weeks old, 25 ± 5 g) were purchased from Hua-Fu-Kang Animal Company (Beijing, China). All animals were housed in temperature-controlled cages with a 12-hour light-dark cycle. This study was carried out in strict accordance with the recommendations in the Guide for the Care and Use of Laboratory Animals of the National Institutes of Health. The animal protocols were reviewed and approved by the Animal Care and Use Committees of General Hospital of Jinan Military District and Qilu Hospital of Shandong University.

In the first part of the animal study, mice were continuously infused with NTG (50 mg/kg/day, 14 days) by planting Alzet osmotic pumps as described previously[[1](#_ENREF_1), [2](#_ENREF_2)] and then mice received MI surgery. At the 14^th^ postoperative day, echocardiography was performed to assess heart function. At the end of experiment, all mice were sacrificed under anesthesia by intraperitoneal injection of 0.8% pentobarbital sodium (60 mg/kg). The heart was harvest to measure Akt activity and S-Nitrosylation.

In the second part of the animal study, mice received tail vein injection of adenovirus expressing wildtype or mutated Akt cDNA. For infection, virus was injected in 100 μl of PBS containing 7.6 × 10^7^ IFUs of loaded lentivirus per. 1 week after virus infection, echocardiography was performed to assess heart function followed by MI surgery and NTG (50 mg/kg/day, 14 days) infusion. At the 14^th^ postoperative day, echocardiography was performed again to assess heart function. At the end of experiment, all mice were sacrificed under anesthesia by intraperitoneal injection of 0.8% pentobarbital sodium (60 mg/kg).

**Cell cultures**

Human umbilical vein endothelial cells (HUVECs) were purchased from Cascade Biologics (Portland, OR) and grown in endothelial basal medium (Clonetics Inc. Walkersville, MD) supplemented with 2% fetal calf serum (FCS) and growth factors, penicillin (100 u/ml), and streptomycin (100 µg/ml). In all experiments, cells were between passages 3 and 8. All cells were incubated at 37°C in a humidified atmosphere of 5% CO_2_ and 95% air. Cells were grown to 70-80% confluent with starvation before being treated with different agents.

**Generation of DNA construct and adenovirus infection to cells or mice**

WT-Akt cDNA were purchased from Origene Company. All cysteine residues were replaced with alanine by using the QuikChange kit (Stratagene), according to the manufacturer's instructions. All mutations were confirmed by DNA sequencing. The adenovirus construction compassing wildtype Akt or mutated Akt was generated using the AdMax (Microbix) and pSilencer™ adeno 1.0-CMV (Ambion) systems according to the manufacturers’ recommendations. Viruses were packaged and amplified in HEK293A cells and purified using CsCl_2_ banding followed by dialysis against 10 mM Tris-buffered saline with 10% glycerol. Titering was performed on HEK293 cells using the Adeno-X Rapid Titer kit (BD Biosciences Clontech, Palo Alto, CA, USA) according to the manufacturer’s instructions. HUVECs were infected with adenovirus overnight in antibiotics-free medium supplemented with 2% FBS. The cells were then washed and incubated in fresh medium for an additional 12-hour before experiments. For infecting mice, adenovirus was injected via tail vein under pressure in 1 ml of PBS with 7.6 X 10^7^ IFUs of loaded virus. The concentration of DNA was 10 mg/kg.

**Determinations of Akt S-nitrosylation and activity**

HUVECs were lysed in HEN buffer (100 mM Hepes, 1 mM EDTA, and 0.1 mM neocuproine, pH 8.0) containing 1% (w/v) SDS and 1 mM PMSF plus protease inhibitors (Hoffmann-La Roche, Basel, Switzerland). The proteins extracted from the HUVECs were quantified using a BCA assay kit (Thermo Fisher Scientific/Pierce). Equal amounts of protein were incubated with HEN buffer containing 2.5% (w/v) SDS and 0.1% (v/v) S-methyl-methanethiosulfonate (Fluka/Sigma-Aldrich) at 50°C in the dark for 20 min. The extracts were precipitated with cold acetone. The proteins were re-suspended in HEN buffer plus 1% SDS. S-nitrosothiols were decomposed by adding ascorbate followed by incubation with 2.5 mg/ml biotin-HPDP (Thermo Fisher Scientific/Pierce) for 1 h at room temperature. The proteins were subsequently precipitated again using acetone and resuspended in non-reducing Laemmli loading buffer. For purification of the biotinylated proteins, the proteins precipitated by acetone were diluted with neutralization buffer [20 mM HEPES (pH 7.7, 100 mM NaCl, 1 mM EDTA, and 0.5% (v/v) Triton X-100] and 50% streptavidin agarose suspension (Fluka/Sigma-Aldrich) and incubated for 1 h at room temperature. The proteins were eluted with elution buffer.

Samples from the biotin switch assay were separated on 12% SDS polyacrylamide gels and transferred to PVDF membranes (EMD Millipore, Billerica, MA, USA). PVDF membranes were blocked with 5% non-fat dried milk for 1 h at 37°C and incubated with specific antibodies, including horseradish peroxidase-conjugated anti-biotin (Cell Signaling Technology, Danvers, MA, USA) overnight at 4°C. Membranes were then incubated with a horseradish peroxidase-conjugated goat against mouse IgG (H+L), followed by enhanced chemiluminescence using the SuperSignal West Pico Substrate kit (both from Thermo Fisher Scientific/Pierce). The protein bands were detected and analyzed using the Chemi-Doc-it HR 410 imaging system (Ultra-Violet Products, Ltd., Upland, CA, USA).

Akt activity was determined in a kinase reaction using recombinant GSK-3α as substrate [[3](#_ENREF_3)]. Two µl GSK-3α Protein/ATP mixture was added into 50 µl kinase buffer and incubated at 30°C for 14 hours. Phosphorylation of the GSK-3α can be analyzed by western blot analysis using the phospho-GSK-3α specific antibody. The level of phospho-GSK-3α was calculated to represent Akt activity.

**Western blot**

Cells or tissues were homogenized on ice in cell-lysis buffer containing 20 mM Tris-HCl (pH 7.5), 150 mM NaCl, 1 mM Na_2_EDTA, 1 mM EGTA, 1% Triton, 2.5 mM sodium pyrophosphate, 1 mM beta-glycerophosphate, 1 mM Na_3_VO_4_, 1 µg/ml leupeptin, and 1 mM PMSF. Protein samples were solubilized in SDS sample buffer, and 20 µg of protein was separated by SDS-PAGE using 8-10% polyacrylamide gels, transferred to nitrocellulose membranes. Entire sheets of Hybond-ECL membranes containing transferred proteins were incubated firstly in 5% non-fat dry milk for 2 hours to block nonspecific binding of antibodies, followed by overnight incubation in primary antibodies diluted 1:1000 at 4°C. The membranes were then washed 3 times with TBST and incubated for 1 hour with second antibody diluted 1:5000 at room temperature. Bound antibodies were detected with ECL-enhanced chemiluminescence (Amersham Biosciences) according to the manufacturer's protocols. Hyperfilm-ECL exposed to membranes for 1 minute was developed in an X-ray film processor. Band intensity (area X density) was measured by densitometry (model GS-700, Imaging Densitometer; Bio-Rad). Background intensity was subtracted from all calculated areas and we used the ratio of control group as 1 as described previously[[4](#_ENREF_4)].

***In vitro* tube formation assay**

The tube formation was performed as the method described previously[[5](#_ENREF_5)]. Cultured HUVEC were seeded on cell culture dishes coated with growth factor reduced Matrigel (BD Biosciences) and cultured in MCDB 133 medium containing 0.5% FCS with or without HG. After 24 hours, the medium was removed and the cells were fixed with 4% paraformaldehyde. Photographs were taken through a microscope (Olympus, Tokyo, Japan). The capillary tube area was quantified per square micrometer using image analysis software (Image J Corporation).

**Cell migration**

Scratch test was applied to detect the migration of cells[[6](#_ENREF_6), [7](#_ENREF_7)]. When the cell growth reached 80% fusion, cell digestion was inoculated into 24 well plates with six duplicated wells for each group. A scratch was made in the well bottom by using a sterile 10 ml spear in cultured cells. The picture of cell migration was taken at day 0 and day 4 after scratch. The migration rate was calculated by counting the distance of cell migrations.

**Evaluation of cell proliferation**

C**ell proliferation was assayed by using** 3-(4,5-dimethyl-2-thiazolyl)-2,5-diphenyl-2-H- tetrazolium bromide (**MTT) as described previously[**[**8**](#_ENREF_8)**]. Cell were grown in a clear plate according to the desired protocol. 50 µL of serum-free media and 50 µL of MTT reagent** (5 mg/ml) **were added into each well. The plate was incubated at 37°C for 3 hours. Then,** dimethyl sulfoxide was added to each well and leave the cells at room temperature in the dark for 2 hours. The absorbance was read at **OD = 590 nm. By averaging the duplicate reading for each sample and subtracting the culture medium background from the assay reading, the amount of absorbance is proportional to cell number.**

**Myocardial infarction**

1. Sterilize surgical instruments with a dry bead sterilizer (Germinator 500).

2. All mice (aged 8-12 weeks) were anesthetized with 2-3% isoflurane inhalation in an inducing chamber.

3. Once anesthetized, the mouse is removed from the inducing chamber to the surgical board, immobilized with tape, and continuously anesthetized with 2% isoflurane via coaxial breathing apparatus but not ventilated.

4. Remove the fur with a standard depilatory (e.g., Nair) and clean the skin with water and then betadine and alcohol pads. In order to perform this procedure more efficiently, the step of fur-removing could be done earlier.

5. Two small incisions (0.5 cm long) are made on the left and right chest skin with the scissors to expose the 3rd intercostal space.

6. Echocardiography is performed using a VEVO 2100 imaging system (Visual Sonics Inc., Toronto, Canada) with a 30 MHz phased array transducer and a frame rate of 235/s. The echocardiography probe (MS-400) is placed perpendicular to the sagittal plane of the chest within the 3rd intercostal space, imaging the left ventricle (LV) short axis.

7. A small straight needle (0.2 mm in diameter) was inserted at the costal angle of the superior margin of the 3^rd^ rib in the left chest. Under the guide of ultrasound, the heart is punctured in the inferior of left anterior descending coronary artery (LADCA) by a 8-0 silk suture attached to the needle. The needle is coming out of skin from the right chest.

8. Then, the needle is inserted back from the right to of the left. When the needle passes through the heart, it goes through above LADCA under ultrasound and came out the skin from the same site in the left chest.

9. Once a loose knot is made, the needle is inserted back from the left to the right in the chest. The LADCA is now located inside of the knot.

10. Ligation of LADCA by pulling the two ends of the suture carefully. The ischemia was confirmed by the elevation of ST segment recorded by the echocardiography imaging system during the surgery. The knot is readily visible under ultrasound.

11. The mouse is then allowed to breathe room air and monitored on a heating blanket during the recovery period, which is generally complete within 3-5 min.

12. The sham group undergoes the same surgical procedures except that the LADCA is not occluded.

13. One dose of buprenorphine (0.1 mg/kg) is administered subcutaneously (s.c.) immediately after the incision is closed.

**Echocardiography**

Echocardiography was performed as described previously[[9](#_ENREF_9)]. Ultrasonography with standard parasternal and apical views was performed in mice in the left lateral recumbent position. We obtained high-quality 2-D images including apical (4-chamber, 2-chamber and long-axis) and short-axis views (mitral annulus, papillary muscle and apex) with use of a 2.0-4.0 MHz transducer at a frame rate of 60 to 100 frames/sec and 3 consecutive cardiac cycles during breath hold. Images were digitized in cine-loop format and stored.

**Capillary Density**

Histological analysis was assessed in perfusion/fixed hearts collected from mice at 14 days after surgery. The right atrium was then cut and the myocardial vasculature was perfused, followed by 10 min perfusion with 10% formalin. The hearts were harvested and fixed in 4% formalin for 24 hours. The formalin-fixed tissues were embedded in paraffin wax and cut into 5 μm sections. For the measurement of capillary density (counts/mm^2^), we performed immunohistochemical analysis of CD31. Transverse sections of the short axis of the left ventricle per sample were used in this analysis. Five fields on the slide were randomly chosen for counting the stained capillaries in the border zone between infarcted area and non-infarcted area at 400X magnification. All stained capillaries were counted, and the density was expressed as number per scope as described previously[[10](#_ENREF_10)].

**Statistical Analysis**

All quantitative results are expressed as mean ± SEM. The normal distribution of data was tested by the Kolmogorov-Smirnov test before statistical comparisons, and the normality/equal variance was tested to determine whether ANOVA was appropriate. Multiple comparisons were analyzed with a one-way ANOVA followed by Tukey *post-hoc* tests or Bonferroni *post-hoc* analyses. Comparisons between two groups were analyzed by unpaired Student's *t* test between two groups. Statistical analyses were conducted using GraphPad Prism 6.0 or IBM SPSS statistics 20.0. A two-sided *P*-value < 0.05 was considered significant.

**References**

[1] Wang S, Zhang C, Zhang M, Liang B, Zhu H, Lee J, et al. Activation of AMP-activated protein kinase alpha2 by nicotine instigates formation of abdominal aortic aneurysms in mice in vivo. Nat Med. 2012;18:902-10.

[2] Li P, Yin YL, Guo T, Sun XY, Ma H, Zhu ML, et al. Inhibition of Aberrant MicroRNA-133a Expression in Endothelial Cells by Statin Prevents Endothelial Dysfunction by Targeting GTP Cyclohydrolase 1 in Vivo. Circulation. 2016;134:1752-65.

[3] Benard L, Oh JG, Cacheux M, Lee A, Nonnenmacher M, Matasic DS, et al. Cardiac Stim1 Silencing Impairs Adaptive Hypertrophy and Promotes Heart Failure Through Inactivation of mTORC2/Akt Signaling. Circulation. 2016;133:1458-71.

[4] Wang S, Xu J, Song P, Viollet B, Zou MH. In vivo activation of AMP-activated protein kinase attenuates diabetes-enhanced degradation of GTP cyclohydrolase I. Diabetes. 2009;58:1893-901.

[5] Nakamura M, Mie M, Mihara H, Nakamura M, Kobatake E. Construction of multi-functional extracellular matrix proteins that promote tube formation of endothelial cells. Biomaterials. 2008;29:2977-86.

[6] Baggott RR, Alfranca A, Lopez-Maderuelo D, Mohamed TM, Escolano A, Oller J, et al. Plasma membrane calcium ATPase isoform 4 inhibits vascular endothelial growth factor-mediated angiogenesis through interaction with calcineurin. Arterioscler Thromb Vasc Biol. 2014;34:2310-20.

[7] Miyake H, Maeda K, Asai N, Shibata R, Ichimiya H, Isotani-Sakakibara M, et al. The actin-binding protein Girdin and its Akt-mediated phosphorylation regulate neointima formation after vascular injury. Circ Res. 2011;108:1170-9.

[8] Zhao H, Zhang T, Xia C, Shi L, Wang S, Zheng X, et al. Berberine ameliorates cartilage degeneration in interleukin-1beta-stimulated rat chondrocytes and in a rat model of osteoarthritis via Akt signalling. Journal of cellular and molecular medicine. 2014;18:283-92.

[9] Yang J, Liu X, Jiang G, Chen Y, Zhang Y, Zhang M. Two-dimensional strain technique to detect the function of coronary collateral circulation. Coron Artery Dis. 2012;23:188-94.

[10] Grundmann S, Hans FP, Kinniry S, Heinke J, Helbing T, Bluhm F, et al. MicroRNA-100 regulates neovascularization by suppression of mammalian target of rapamycin in endothelial and vascular smooth muscle cells. Circulation. 2011;123:999-1009.
